# Meta-Optics-Empowered Switchable Integrated Mode Converter Based on the Adjoint Method

**DOI:** 10.3390/nano12193395

**Published:** 2022-09-28

**Authors:** Yingli Ha, Yinghui Guo, Mingbo Pu, Mingfeng Xu, Xiong Li, Xiaoliang Ma, Fang Zou, Xiangang Luo

**Affiliations:** 1 State Key Laboratory of Optical Technologies on Nano-Fabrication and Micro-Engineering, Institute of Optics and Electronics, Chinese Academy of Sciences, Chengdu 610209, China; 2 University of Chinese Academy of Sciences, Beijing 100049, China; 3 Tianfu Xinglong Lake Laboratory, Chengdu 610299, China

**Keywords:** switchable mode converter, adjoint method, meta-optics, photonic integrated circuits

## Abstract

Monolithic integrated mode converters with high integration are essential to photonic integrated circuits (PICs), and they are widely used in next-generation optical communications and complex quantum systems. It is expected that PICs will become more miniaturized, multifunctional, and intelligent with the development of micro/nano-technology. The increase in design space makes it difficult to realize high-performance device design based on traditional parameter sweeping or heuristic design, especially in the optimal design of reconfigurable PIC devices. Combining the mode coupling theory and adjoint calculation method, we proposed a design method for a switchable mode converter. The device could realize the transmission of TE0 mode and the conversion from TE0 to TE1 mode with a footprint of 0.9 × 7.5 μm^2^ based on the phase change materials (PCMs). We also found that the mode purity could reach 78.2% in both states at the working wavelength of 1.55 μm. The designed method will provide a new impetus for programmable photonic integrated devices and find broad application prospects in communication, optical neural networks, and sensing.

## 1. Introduction

As an emerging method of interconnection, photonic integrated circuit (PIC) interconnections have many incomparable advantages for electric interconnection, such as extremely high bandwidth, an ultra-fast transmission rate, and high anti-interference resistance, which provide the necessary technical means to break through the bottleneck of traditional microelectronic chip interconnection [1]. In 2018, David R. Smith proposed “subwavelength integrated photonics”, which is expected to become the core of the next generation of PICs [2]. Almost at the same time, our team proposed the concept of the chip-integrated metasurface as a novel platform for directional coupling [3], varifocal waveguide lens [4] and holographic systems [5], beam scanning [6], and crosstalk suppression [7]. The appearance of subwavelength artificial structures (SAS) has brought a new degree of freedom to the design of traditional PICs, which can improve the performance of devices and reduce the footprint of devices by enhancing the interaction strength of light and matter [8]. A device with a length of 10 wavelengths can realize mode conversion and its efficiency is as high as 90% [9]. In addition, using the optimization algorithm will help to further improve the efficiency and integration of sub-wavelength PICs [10,11,12,13,14].

One of the obstacles on the road to subwavelength PIC application is the fact that static subwavelength PICs cannot solve dynamic information transmission [10,11,15,16]. Phase change materials (PCMs) [17,18] and all-optical [14,19], electro-optical [20,21], and thermo-optical [22] modulation could realize the modulation of the material refractive index. PCMs are widely used in tunable meta-optics due to the great difference in refractive index between the two states, even though the modulation speed and energy consumption are slightly inferior compared with other modulation methods. Electricity [17,18], heat [23,24], and light [25] stimulation can be applied in PCMs to change the wavefront passing through the meta-optics, such as optical switches [26,27], optical routers [28], and switchable meta-optics [29,30,31]. A mode conversion device based on PCMs can realize different types of mode conversion and provide extra physical dimensions for multiplexing optical information, enabling applications such as encrypted holography [32], high-throughput communication [32,33], ultrafast modulation [34], high-efficiency optical calculation [26,35,36], etc.

As an efficient optimization algorithm, each iteration optimization can only be achieved through one forward simulation and one adjoint simulation. Compared with other optimization algorithms, this requires a great deal of parallel optimization and optimization to update the gradient, which will greatly reduce the calculation time. A freeform structure has better performance [37]. For a freeform structure and multi-objective optimization problem, the particle swarm optimization algorithm [7,38], genetic algorithm [39,40], and other traditional optimization algorithms [16,41] consume a great deal of computing resources and time due to their high design dimensions. Compared with the traditional algorithms mentioned above, topology optimization has great advantages in the design of micro-/nanostructures. There are two specific reasons. Firstly, the traditional algorithm depends on the initial structure. In most cases, the initial structure is based on existing theoretical models, and a completely random initial structure may not be efficient. As with the DBS algorithm, the result depends on the random initial structure and also depends on the randomly optimized pixels. The topology optimization algorithm is a full-model structure optimization, which only depends on the initial structure. In contrast, we use a completely random initial structure, which is less likely to fall into local optima. Secondly, in an iterative optimization, topology optimization only requires one forward and one reverse calculation. However, the DBS algorithm needs to traverse all pixels in an iterative optimization. In contrast, the calculation amount of our method is two orders of magnitude smaller than other traditional algorithms. Above all, the topology optimization algorithm obtains the freeform structure distribution by designing a high design freedom space, which could realize high integration and high efficiency at the same time. In addition, the topology optimization algorithm can be used to realize multi-dimensional meta-optics lacking theoretical models, such as mode beam splitting [16,42], wavelength beam splitting [10], and other devices [11,43].

In this paper, we propose a high-integration programmable mode converter based on Ge_2_Sb_2_Te_5_ (GST-225). Using electric gating, GST-225 can produce reversible and relatively stable conversion between amorphous and crystalline states to achieve significant changes in electromagnetic parameters and adjust the function of the device. Combining the tremendous differences between crystallization and amorphous states, a mode converter could realize TE0 mode transmission and TE0 to TE1 mode conversion. The results show that, through the adjoint optimization method, we can further improve the error between the optical response of the cell structure obtained by the periodic boundary conditions and the actual results. The integration of the device is 0.9 × 7.5 μm^2^, which is defined as the area of the GST-225 material. In the bandwidth range of 1.4 to 1.7 μm, the device can realize average transmission efficiency of 70% for TE0 mode and mode conversion efficiency of 80% for TE0 to TE1 mode. The proposed mode converter device has a wide range of applications in optical computing [44,45], communication [45], optical neural networks [46,47], etc. Furthermore, the design method can be used to improve the performance and integration of meta-optics. The designed programmable PICs prove that artificial intelligence and meta-optics in synergy will aid the research and development of advanced optical chips.

## 2. Materials and Methods

**Figure 1:**
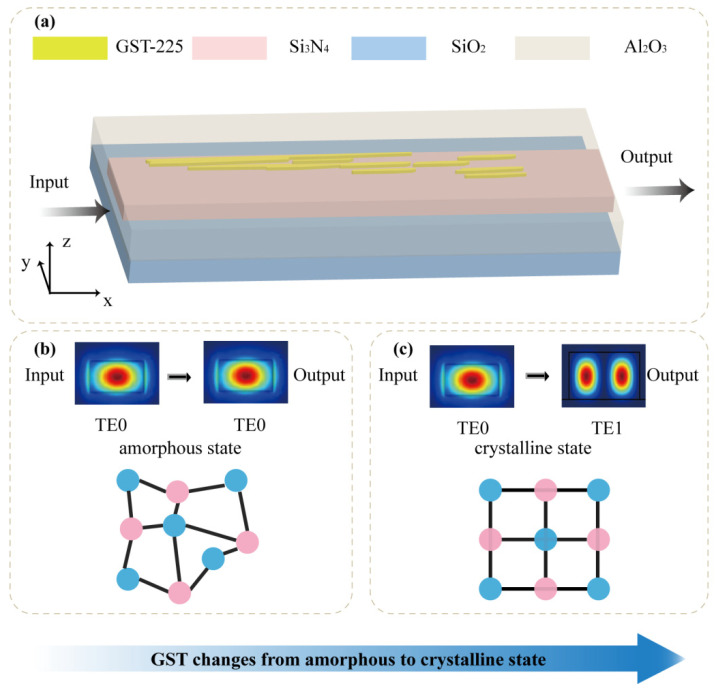
The principles of guided wave-driven metasurfaces. (**a**) The schematic of the programmable mode converter. Inset: cross-section view of the mode converter. (**b**) The electric field distribution on input and output port, when GST-225 is in the amorphous state. (**c**) The electric field distribution on input and output port, when GST-225 is in the crystalline state.

Figure 1a shows a 3D schematic of the programmable mode converter, which consists of a freeform GST-225 metasurface directly integrated on a silicon nitride (Si_3_N_4_) waveguide. A subwavelength structure and GST-225 material are used to change the propagation constants in waveguides. When GST-225 is in the amorphous state, the permittivity is 4.725 + 0.07i, and when GST-225 is in the crystalline phase, the permittivity is 7.49 + 1.356i [26]. Since the imaginary part of the refractive index of GST-225 cannot be ignored, we set its thickness to 30 nm to reduce the transmission loss. Comparing the extinction coefficients, when GST-225 is in the crystalline state, its value is nearly 20 times that of the amorphous state. In terms of device loss, the efficiency of amorphous devices is higher than that of crystalline devices, and the difference in efficiency is related to the structure distribution. The efficiency effect caused by the difference in extinction coefficient will be given later. To ensure high-transmission efficiency of TE0 and TE1 modes in the waveguide, the thickness and width of the waveguide are 0.33 μm and 1.8 μm, respectively. A layer of Al_2_O_3_ material with a dielectric constant between the two materials is added between the waveguide layer and the air, which not only improves the mode transmission efficiency of the waveguide but also improves the integration. Referring to the previous literature [26], we set its thickness to 218 nm and the permittivities of Si_3_N_4_ and Al_2_O_3_ are 1.7 and 1.74. Figure 1b,c illustrate that when the GST-225 is in the crystalline state, the waveguide realizes single-mode transmission of the TE0 mode. Because the GST-225 material can compensate for the propagation constant difference between TE0 and TE1 modes, the device realizes the conversion of the TE0 mode to the TE1 mode when the GST-225 changes from the crystalline state to the amorphous state.

**Figure 2:**
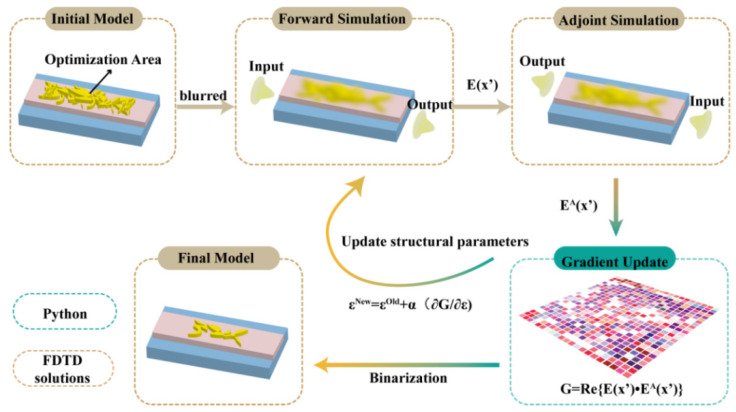
Design flow diagram.

It is necessary to use the physical-driven adjoint simulation optimization algorithm to update the entire device structure and improve the design efficiency of meta-optics through only one forward and one adjoint simulation due to the high computational costs of the current design methods. In addition, previous single software simulations still display some problems—for instance, the imperfect theoretical model of complex functions, and the cumbersome process of modifying structures. The intelligent inverse design method can automatically modify complex device structures by interconnecting programmable software and electromagnetic simulation software. As shown in Figure 2, the adjoint simulation calculation is carried out by combining programmable software—Pycharm—and electromagnetic simulation software—FDTD Solutions. Through the real-time transmission of simulation results, the accuracy of complex structure design is improved, and the cumbersome structure correction is simplified. Due to the different wave vectors of different modes, the GST structure acts as a phase shift device. If the GST structure can compensate for the wave vectors between different modes, mode conversion would be realized between the two modes. By calculating the equivalent refractive index *n*_1_ and *n*_2_ of TE0 and TE1 modes at the working wavelength of 1550 nm, the propagation constants are obtained, respectively. According to the propagation constant difference between different modes, we can roughly determine the size of the optimization region. This is important for design without prior knowledge, because determining the simulation area involves reducing the optimization parameters in order to save computing resources. In our design, *n*_1_ and *n*_2_ are 1.7 and 1.63, respectively.

Firstly, a randomly distributed initial photonic integrated device is generated in the design area, and the randomly generated structure is a binary distribution structure of 1 and 0, which means that it either has GST-225 material or is without GST-225 material. We refer to the previous work and position the size of the optimized area at 1.8 × 10 μm^2^ [26], which is divided into 1000 × 200 pixels. Secondly, the structure is blurred to ensure that its dielectric constant is between those of Al_2_O_3_ and SiN. The FDTD Solutions software is used to simulate the structure through the finite-difference time-domain method, and its forward field and adjoint field are obtained. Regardless of the crystal state of the GST-225, in the forward simulation, the simulated wavelength is 1.55 μm with TE0 mode. In adjoint optimization, its companion source is the distribution of the ideal target light field. Specifically, when the GST-225 is in the crystalline state, the adjoint source is the TE0 mode, and when the GST-225 is in the amorphous state, the adjoint source is the TE1 mode. Then, the gradient of the objective function is calculated by programmable software, and the spatial distribution of the refractive index of the structure is updated. The figure of merit (FoM) is defined as the integral of all the targets in the objective domain as in Equation (1):

(1) $$F(E,H)=\int_{D0}f(E(x_{0}),H(x_{0}))d^{3}x_{0}$$

where *E* and *H* are the actual EM fields at the *x*_0_ position from the simulation. The derivative of total FoM in the objective domain to permittivity *ε* in the design domain is shown in Equation (2):

(2) $$G\left(x_{S}\right)=2Re[E(x_{S})E^{A}(x_{S})]$$

where *E^A^* is the adjoint electric field and can be obtained through the integration of the electric fields calculated by the FDTD Solutions software. The permittivity in the design domain is updated towards the direction of total gradient descent as in Equation (3):

(3) $${\varepsilon_{S}}^{new}={\varepsilon_{S}}^{old}+\alpha G$$

where *ε*_s_*^new^* is the new permittivity distribution after one iteration, and *ε*_s_*^old^* is the old permittivity distribution. *α* is the updating rate. In each iteration, the permittivities of all pixels are updated to a better solution by the new gradient of the previous generation. Through iterative training, the FoM function converges and the final optimization result is obtained.

## 3. Results

**Figure 3:**
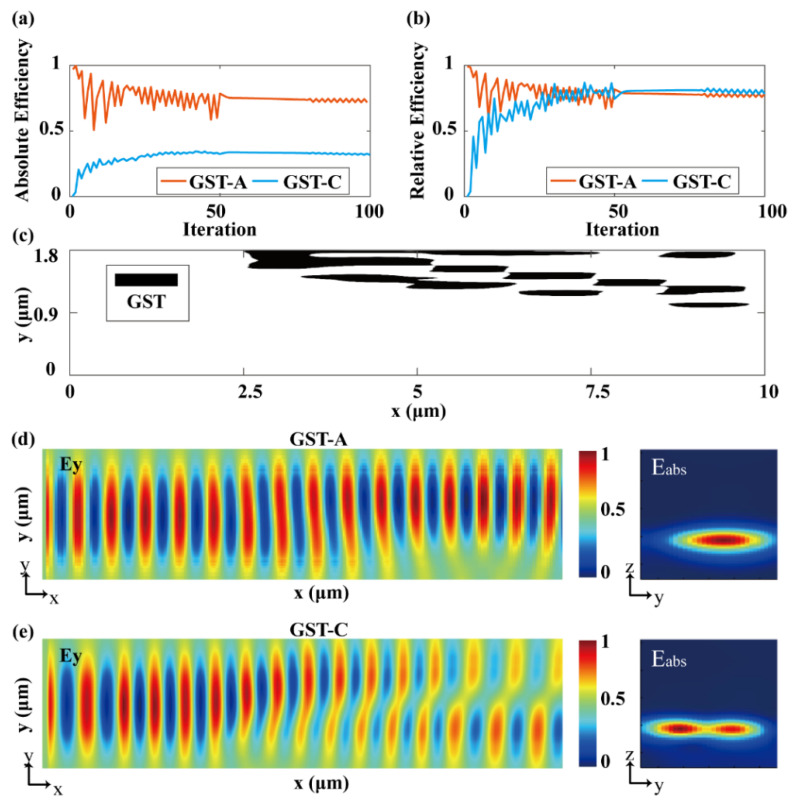
(**a**) The evolution of the transmission efficiency with the number of iterations when GST-225 is in the crystalline and amorphous states. (**b**) The evolution of the relative transfer efficiency of GST-225 with the number of iterations when it is in the crystalline and amorphous states. (**c**) Schematic diagram of the final optimized structure distribution. (**d**) When GST-225 is in amorphous state, TE mode is transmitted in the forward and backward propagation direction. (**e**) When GST-225 is in crystallization state, TE mode is converted to TE1 mode in the forward direction, and TE1 mode is converted to TE0 mode in the backward direction.

To realize mode conversion, several mature techniques used in the above works can be adopted. By applying a specific electrical stimulus, the phase state can be changed among different states, thus realizing mode transmission and conversion [29]. The second method is based on the direct writing of a femtosecond (fs) laser [48]. The re-amorphization of the GST-225 layer can be achieved by a single pulse with high power intensity, and the crystallization of the GST-225 layer can be achieved by a pulse sequence with lower power intensity. As shown in Figure 3a, the initial absolute efficiency of the device in the two crystal states is 0% and 97%, respectively. It can be seen that in the first 10 generations, the efficiency of TE0 mode and TE1 mode changes rapidly, and the evolution rises slowly after 20 iterations. After 200 iterations, the absolute efficiency of TE1 mode in the crystallization state is improved from 0% to 31.6%. The transmittance of TE0 mode in the amorphous state is 70%. We have realized the functions of mode transmission and mode conversion based on an adjoint optimization algorithm. Compared with the switchable mode converter reported before [26], the transmission is improved from 24% to 31.6%, and the relative efficiency is increased by 31.7%. As we can see, the transmission efficiency of the mode converter device in the crystalline state is lower than that in the amorphous state, which is due to the difference caused by the loss of the GST-225 material in the amorphous and crystalline states. The absolute efficiency also represents the evolution of the mode purity with the number of iterations, as shown in Figure 3b. In the initial state, the relative efficiencies of the device in the two states are 0% and 99.8%, and after 200 iterations, the above values reach 78.2%. The mode purity of TE0 mode and TE1 mode in the two states is calculated by the following equation:

(4) $$\eta =\frac{1}{n}\sum_{\lambda =i}^{n}\frac{T_{TE0/TE1}}{T_{TE1}+T_{TE0}+\ldots +T_{TEm}}$$

where *η* is the mode purity and *T* is the transmission of *TE*0, *TE*1, and other higher-order modes. *n* is the number of working wavelengths. The final structure distribution is shown in Figure 3c, which is basically distributed at 2.5~10 μm and 0.9~1.8 μm on the *x-* and *y-*axes. The optimized GST-225 length is 7.5 μm, which is an order of magnitude lower than the length of the regular-shaped mode converter that has been reported in Ref. [26].

To verify the performance of the final optimized structure, we conducted a full model simulation and obtained the electric field distribution on the *xy* plane, as shown in Figure 3d,e. Here, we define “forward” as the propagation direction of the optical waveguide mode along the *x* direction in Figure 1a, and “reverse” as the propagation direction of the waveguide mode along -*x*. It can be seen from the electric field that when GST-225 is in the amorphous state, the mode distribution of TE0 mode in the waveguide is not affected by the structure of GST-225, except for the electromagnetic loss. As shown in Figure 3e, when GST-225 is in the crystalline state, the perturbation TE0 mode receiving the freeform GST-225 structure gradually changes from 4 μm to TE1 mode, accompanied by a large amount of energy loss. Figure 3d,e show the total electric field distribution in the *yz* plane on the left side of the device, from which we can clearly see that they are TE0 mode and TE1 mode, respectively.

**Figure 4:**
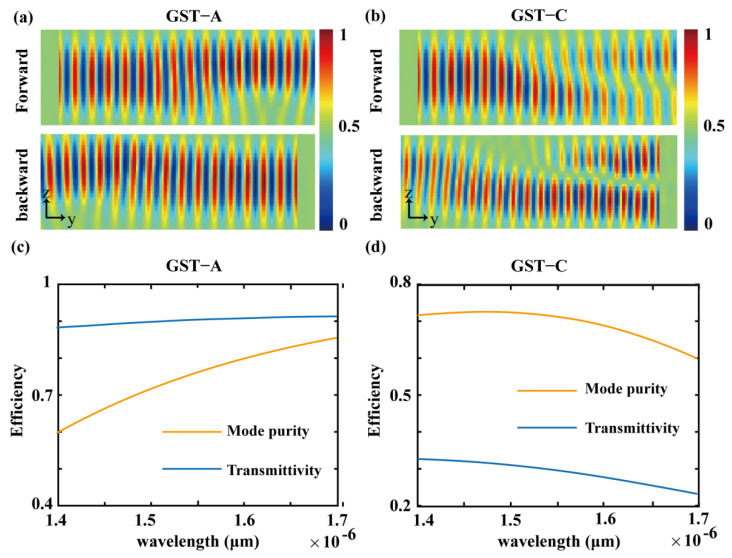
(**a**) When GST-225 is in the amorphous state, TE0 mode can be output in TE0 mode at the forward or backward transmission. (**b**) When the GST-225 is in the crystalline state, the TE0 mode and the TE1 mode are transmitted in the forward and backward transmission, and another mode is obtained at the output. (**c**,**d**) When GST-225 is in the amorphous and crystalline state, the transmission efficiency and mode purity distribution differ at different wavelengths.

It should be noted that this directional waveguide mode coupling will not break the time-reversal symmetry. Figure 4a,b show finite-difference time-domain simulations describing the evolution of the modes in two opposite propagation directions. When GST-225 is in the amorphous state, for the forward or backward input, TE0 mode is transmitted from input to output. Conversely, when GST-225 is in the crystalline state, if TE0 mode is started along the forward direction, it will be effectively converted to TE1 mode upon exiting from the output port. If TE1 mode is started in the backward direction, it will be effectively converted to TE0 mode upon exiting from the output port. In order to verify the broadband effect of the designed device, we simulated and verified the mode conversion of 10 nm from 1.40 μm to 1.70 μm, and results are shown in Figure 4c,d. The optical power transfer spectrum of the device in the direction of propagation and the ratio of the spectrum indicate that efficient optical power transfer is maintained over a wide wavelength range. From 1.40 μm to 1.70 μm, the mode purity is maintained at around 70%. The transmission of TE0 and TE1 modes is maintained at around 30% and 90%. 

## 4. Conclusions

In summary, we have modeled a monolithic-integrated programmable mode converter based on GST-225 material. Using a random distribution GST-225 as the initial structure, we designed a tunable mode converter that could realize TE0 mode transmission and TE0 to TE1 mode conversion for amorphous and crystallization states. According to the topology optimization algorithm, the performance and integration of the mode converter have been well improved. Breaking the previous topology optimization applied to the design of static meta-optics, we can realize the optimization design of dynamic tunable devices. The results show great potential for the design of next-generation SAS-based high-integration PICs. Although it is difficult to realize machining technology for devices of arbitrary shape, efficient and high-precision processing can be achieved simultaneously by appropriately compensating for and removing some structures with smaller feature sizes. With the gradual improvement of micro-/nano-technology, the fabrication of meta-optics with arbitrary freeform structures is imminent. As PICs gradually become a general technology platform, phase-change photonic devices can further expand the application of integrated photonic chips in data center optical interconnection [44], nonlinear and quantum photonics [49], and on-chip optical sensors [50]. Besides the mode converter, the optical resonators, modulators, and demultiplexers can be optimized by this method. In addition, the use of highly integrated silicon materials and low-loss phase change materials is conducive to the design of photonic devices with higher performance and higher integration levels.

## Data Availability

The data presented in this study are available in this article.
